# Sutureless Repair for Open Treatment of Inguinal Hernia: Three Techniques in Comparison

**DOI:** 10.3390/jcm13020589

**Published:** 2024-01-19

**Authors:** Enke Baldini, Eleonora Lori, Carola Morini, Luigi Palla, Diego Coletta, Giuseppe M. De Luca, Giorgio Giraudo, Sergio G. Intini, Bruno Perotti, Angelo Sorge, Giampaolo Sozio, Marco Arganini, Elsa Beltrami, Daniele Pironi, Massimo Ranalli, Cecilia Saviano, Alberto Patriti, Sofia Usai, Nicola Vernaccini, Francesco Vittore, Vito D’Andrea, Priscilla Nardi, Salvatore Sorrenti, Piergaspare Palumbo

**Affiliations:** 1 Department of Surgery, “Sapienza” University of Rome, 00161 Rome, Italy; enke.baldini@uniroma1.it (E.B.); eleonora.lori@uniroma1.it (E.L.); morini.1743951@studenti.uniroma1.it (C.M.); daniele.pironi@uniroma1.it (D.P.); usaisofia13@gmail.com (S.U.); vito.dandrea@uniroma1.it (V.D.); priscilla.nardi@uniroma1.it (P.N.); salvatore.sorrenti@uniroma1.it (S.S.); 2 Department of Public Health and Infectious Diseases, “Sapienza” University of Rome, 00161 Rome, Italy; luigi.palla@uniroma1.it; 3 United Hospitals of Northern Marche (AOORMN)—Pesaro, 61121 Pesaro, Italy; diegocoletta1@gmail.com (D.C.); albertopatriti@gmail.com (A.P.); 4 Unit of Academic General Surgery “V. Bonomo”, University of Bari, 70124 Bari, Italy; max-de-luca@libero.it (G.M.D.L.); f_vittore@yahoo.it (F.V.); 5 Department of Surgery, Santa Croce e Carle Hospital (ASO) of Cuneo, 12100 Cuneo, Italy; dr.giorgiogiraudo@gmail.com (G.G.); elsabeltrami@hotmail.it (E.B.); 6 Department of Surgery, S. Maria Della Misericordia Hospital, ASUFC of Udine, 33100 Udine, Italy; sergio.intini@uniud.it (S.G.I.); nicola.vernaccini@asufc.sanita.fvg.it (N.V.); 7 Department of Surgery, Versilia Hospital of Viareggio, 55049 Camaiore, Italy; bruno.perotti86@gmail.com (B.P.); marco.arganini@uslnordovest.toscana.it (M.A.); 8 Day Surgery P.O.S. Giovanni Bosco, 80144 Naples, Italy; ansorge@alice.it (A.S.); ceciliadoc@virgilio.it (C.S.); 9 Department of Surgery, Alta Val D’Elsa Hospital of Poggibonsi—Siena, 53036 Poggibonsi, Italy; giampaolo.sozio@uslsudest.toscana.it (G.S.); massimo.ranalli@uslsudest.toscana.it (M.R.)

**Keywords:** inguinal hernia, sutureless repair, postoperative complications, antithrombotic therapy

## Abstract

Currently, groin hernia repair is mostly performed with application of mesh prostheses fixed with or without suture. However, views on safety and efficacy of different surgical approaches are still partly discordant. In this multicentre retrospective study, three sutureless procedures, i.e., mesh fixation with glue, application of self-gripping mesh, and Trabucco’s technique, were compared in 1034 patients with primary unilateral non-complicated inguinal hernia subjected to open anterior surgery. Patient-related features, comorbidities, and drugs potentially affecting the intervention outcomes were also examined. The incidence of postoperative complications, acute and chronic pain, and time until discharge were assessed. A multivariate logistic regression was used to compare the odds ratio of the surgical techniques adjusting for other risk factors. The application of standard/heavy mesh, performed in the Trabucco’s technique, was found to significantly increase the odds ratio of hematomas (*p* = 0.014) and, most notably, of acute postoperative pain (*p* < 0.001). Among the clinical parameters, antithrombotic therapy and large hernia size were independent risk factors for hematomas and longer hospital stay, whilst small hernias were an independent predictor of pain. Overall, our findings suggest that the Trabucco’s technique should not be preferred in patients with a large hernia and on antithrombotic therapy.

## 1. Introduction

Inguinal hernia is a very common condition worldwide that occurs when part of the abdominal content protrudes through a weak area of the transversalis fascia in the groin region. In adults, hernias that expand cause discomfort or become strangulated, and are treated by surgery (hernioplasty or herniorrhaphy). Repair procedures have changed radically over time, from techniques such as Bassini’s—consisting in a surgical reconstruction of the inguinal canal—to the current tension-free and sutureless approaches entailing the application of prostheses usually performed on an outpatient basis [1,2,3]. Lichtenstein’s mesh repair, introduced in 1984, has been the most used technique for inguinal hernias until a few years ago. Despite being safe and highly effective, the original Lichtenstein’s technique includes a mesh fixation with non-absorbable suture to prevent displacement or wrinkling, which results in increased postoperative chronic pain [4,5].

In 1989, Trabucco proposed a tension-free, sutureless inguinal hernioplasty that employs a flat plug and a pre-shaped mesh. The latter is placed into the inguinal box between the transversalis fascia and the aponeurosis of the external oblique, where it remains stable without suture points. [6]. Other sutureless methods of mesh fixation have been devised over the years which make use of tacks, staples, fibrin, cyanoacrylate glues, or self-fixing mesh.

Following the increasingly widespread use of different approaches, a series of studies have been aimed at evaluating their effectiveness in definitive hernia repair while ensuring a good postoperative course and a quick return to normal daily activities. In the context of the open hernioplasty, a number of works have compared performances of suture and sutureless techniques, with the data combined by several systematic reviews and meta-analyses (SRMAs), especially with regard to glue versus suture [7,8,9,10,11] and self-gripping versus suture [12,13,14,15,16,17,18,19]. Recently, an umbrella review was conducted to pull together data from previous SRMAs concerning both open and laparoscopic hernia repair [20]. The conclusion derived from this analysis was that glue and self-gripping mesh were effective in reducing chronic groin pain and operating time, respectively, with class III level of evidence.

Overall, the advantages of using non-suture methods have been recognized by most studies [21]. Nonetheless, the most recent international guidelines for groin hernia management did not indicate a gold standard procedure for hernioplasty, but rather suggested tailoring the surgical treatment to specific cases based on the surgeon’s expertise, hernia type, and patient’s characteristics [22]. Furthermore, there is still no agreement on the convenience of using one sutureless fixation method over another, and, additionally, sutureless methods are not yet universally accepted. A possible reason for this discordance is that the postoperative course, in addition to the surgical techniques and type of mesh applied, depends on factors such as patient’s gender, age, state of health, and drug regimen, which are not always taken into consideration or are evaluated unevenly across studies.

On this basis, the present research was aimed at comparing patients subjected to open hernia repair with three different sutureless procedures, i.e., the Trabucco’s technique, mesh fixation with fibrin glue, and use of self-gripping mesh, to assess the incidence of minor and major postoperative complications, acute and chronic pain, and hospital stay duration. Potential effects of patient-related features, such as age, comorbidities, use of antithrombotic drugs, ASA score, hernia location and size, on the outcomes were also examined.

## 2. Materials and Methods

### 2.1. Study Design

This is a multicentre retrospective study involving eight Italian surgical units that was conceived and conducted referring to the STROBE statement [23]. Before starting data collection, surgeons from the various teams held meetings to discuss the surgical techniques under examination and any information available in their respective archives to build a standardized and homogeneous database.

In total, 3920 records of patients subjected to surgery for inguinal hernia between January 2015 and January 2019 were screened, and 1034 patients were enrolled according to the following eligibility criteria: age ≥ 18 years; primary unilateral non-complicated inguinal hernia; ASA (American Society of Anesthesiologists) score 1–2, and ASA score 3 with stabilized disease; open anterior sutureless repair; surgical procedures carried out in accordance with the selected techniques.

The use of patients’ medical records for research purposes respected the ethical standards of the Helsinki Declaration of 1975 and was approved by the Ethical Committees of the competent hospitals.

### 2.2. Patients’ Data

Inguinal hernias were grouped based on the classification proposed by the European Hernia Society (EHS) [24]. Specifically, hernia locations were distinguished in lateral/indirect (L), medial/direct (M), and latero-medial (LM), and orifice sizes were classified as 1, 2, or 3 based on whether it was possible to insert 1, 1–2, or 3 fingers, respectively. For each patient, age, gender, comorbidities, use of antiplatelet/anticoagulant therapy, and ASA (American Society of Anesthesiologists) score [25] were collected. There were few missing data points limited to hernia size and location, as well as the ASA score (see Table 1), which were randomly distributed among the patient groups.

Among the various comorbidities noticed, only those that could influence the patient’s postoperative course, both in terms of complications and sensitivity to pain, were considered. These were classified into five major groups: cardiovascular diseases (e.g., hypertension, cardiomyopathies, aortic aneurysm, cardiac insufficiency, phlebopathy, and peripheral arteriopathy), metabolic diseases (e.g., diabetes, hepatic steatosis, chronic liver disease, and cirrhosis), respiratory diseases (e.g., chronic obstructive pulmonary disease, bronchial asthma), preexisting infections (e.g., HIV, hepatitis), and autoimmune diseases (e.g., multiple sclerosis, systemic lupus erythematosus, Sjögren’s syndrome, and rheumatoid arthritis). Other diseases not falling into the above groups but present only in a few cases were not considered. The perioperative management of antithrombotic therapies was performed according to the American College of Chest Physicians Clinical Practice guidelines [26].

In all the facilities involved in the study, scheduled follow-up appointments included controls on the 7th and 30th days, and an in-person or a phone follow-up after 1 year. Further visits during the first year after the operation were carried out with timing based on the patients’ needs, but they were not taken into consideration as this information was much more heterogeneous.

### 2.3. Outcomes

The primary outcome of the study was the comparison of different sutureless techniques of hernia repair with regard to patient safety and wellness. In particular, the occurrence of surgery related complications, i.e., wound infection, hematoma, scrotal edema, and seroma in a time frame of 30 days were assessed, as well as the onset and evolution of posthernioplasty pain and the frequency of recurrence in the following year. The postoperative acute pain was quantified with the Numerical Rating Scale (NRS) on the 7th and 30th days after surgery, and chronic pain was checked after 1 year.

The secondary outcome of the study was the comparison of postoperative hospital stay durations with respect to patients subjected to the different surgical techniques. All patients were discharged within 12 h (day surgery) or after 16 h. Therefore, the time until discharge was dichotomized based on the 12 h cut-off value so as to distinguish outpatients and inpatients.

### 2.4. Surgical Techniques

Patients were divided into three groups according to the sutureless technique employed, namely Trabucco’s repair, the use of self-gripping mesh (ProGrip^®^ Medtronic, Minneapolis, MN, USA), or the use of fibrin sealant (Tisseel/Tissucol^®^ Baxter, Deerfield, IL, USA), chosen by surgeons based on their expertise and operating preferences. All patients underwent local anesthesia. When the repair was accomplished with the Trabucco’s technique, surgeons used a semi-rigid flat pre-shaped polypropylene mesh combined with a polypropylene plug. In other interventions, the mesh was fixed with a fibrin glue (Tisseel) dispensed by a needle or a spray applicator according to the surgeons’ preference and confidence. Hernioplasty with the ProGrip mesh was performed by using the mesh either alone (n = 91) or together with an absorbable hemostatic gauze (Tabotamp^®^ (Johnson & Johnson Medical, Ethicon, Neuchâtel, Switzerland)) (n = 221). These two subgroups of patients were compared in preliminary statistical analyses, and the results obtained showed no significant differences for any of the outcomes of interest. Therefore, all patients receiving ProGrip were considered as a single group in the subsequent statistical tests.

The meshes were classified into two major categories based on weight, that is light and standard/heavy [27]. Prostheses used in the Trabucco’s technique were standard/heavyweight, while the others were lightweight.

During all the procedures, surgeons identified and preserved ilioinguinal, genital branch of genitofemoral, and iliohypogastric nerves [28].

### 2.5. Statistics

At first, bivariate analyses were carried out to establish which patients’ characteristics were associated to complications (infection, hematoma, scrotal edema, and seroma), postsurgical acute or chronic pain, and time until discharge. Bivariate analyses were also performed to find significant associations between patients’ features and intervention types. The latter results were used to select variables for the subsequent multivariate analysis as explained below.

In all statistical tests, pain was dichotomized into yes/no categories, and surveys conducted on the 7th and 30th days were merged into the variable “presence/absence of pain within the first month”. Hematoma, scrotal edema, seroma, and infection were analyzed separately from each other. Chi-square or Fisher exact tests (when ≥20% of cells of the contingency table had expected frequencies < 5) were applied to categorical variables, followed by the *z*-test for independent proportions with the Bonferroni correction for multiple comparisons. The Mann–Whitney test was used to assess whether patients with complications, pain, or time until discharge > 12 h differed significantly by age.

Logistic regression analyses were performed to estimate how the surgical techniques affected hematomas, acute postoperative pain, or time until discharge. Other complications (seroma, scrotal edema, and infection) and chronic pain were not suitable for this analysis because of the small number of events. The dataset of independent variables comprised surgical treatment and the covariates which were found to associate with both treatment and outcome in the bivariate analysis (potential confounders), considering a threshold *p*-value ≤  0.10. Due to the high correlation between intervention and mesh weight, a separate analysis was carried out with the same criteria but replacing the intervention with the mesh weight. All statistical analyses were conducted using the SPSS software for Windows v.28 (SPSS, Inc., Chicago, IL, USA).

## 3. Results

### 3.1. Patients’ Description

All potentially relevant information on patients that could be drawn from medical records are summarized in Table 1. Cardiovascular diseases were the most common comorbidities, among which hypertension (with or without concurrent pathologies) was present in 315 out of 351 cases. Chronic obstructive pulmonary disease appeared in 55 out of 77 patients affected by respiratory diseases, and type I or II diabetes was encountered in 58 out of 69 cases of metabolic disorders. No major intra- or postoperative complications (e.g., myocardial infarction, hemorrhage, thromboembolic events, etc.) occurred in patients. The time until discharge exceeded 12 h in 151 cases (14.6%) and 48 h in 19 (1.8%). On the whole, hematoma was the most frequent postoperative complication with 67 cases (6.5%) observed, followed by seroma (n = 11; 1.1%), infection (n = 11; 1.1%), and scrotal edema (n = 5; 0.5%).

Within the first year after surgery, eight patients (0.8%) experienced recurrence, of which four had been operated on with the Trabucco’s technique, two with the application of ProGrip, and two with the use of Tisseel. Recurrences were not significantly associated with any patient characteristics, postoperative complications, surgical technique, or type of mesh applied.

### 3.2. Bivariate Analysis of Postoperative Complications

In the bivariate analysis, hematomas were significantly associated with the male gender, older age, hernia size 3, ASA score 3, antithrombotic therapy, presence of comorbidities, cardiovascular, metabolic, and autoimmune diseases, and use of standard/heavy mesh, but did not differ among hernia repair techniques (see Table 2). With regard to other postsurgical complications, no correlations with clinical features emerged, probably due to their low rate. Scrotal edemas were significantly associated with the application of standard/heavy mesh, while all postoperative infections occurred after Trabucco’s repair. Seroma appeared not to be influenced by any of the parameters examined.

### 3.3. Bivariate Analysis of Postoperative Acute Pain and Time to Discharge

Postoperative acute pain was reported by 175 (16.9%) patients on the 7th day, and by the 20th (1.9%) and 30th day. When dichotomized as presence/absence of the symptom within 30 days, pain was reported by a total of 181 (17.5%) patients. After 1 year, six of them still had pain, while three patients with no postoperative acute pain developed neuralgia. Of the six patients with both acute and chronic pain, two had been operated on with Trabucco’s technique and four with ProGrip; the remaining three had undergone hernia repair with Tisseel. Overall, the intensity of chronic pain reported by patients was mild, the mean NRS score being 1.78 ± 0.8 with a maximum value of 3.

As expected, a positive correlation was found between postoperative acute pain and presence of hematoma or scrotal edema. However, the onset of pain was inversely related to the hernia orifice width (see Table 3). Among patients who felt pain 7 days after surgery, the mean NRS score was higher in those with size 1 hernia than in those with size 2 hernia (2.9 ± 1.9 vs. 2.0 ± 1.3, *p* = 0.01), while it did not differ significantly between patients with size 1 and 3 hernias. This difference was no longer evident on day 30 or after 1 year. In addition, acute pain was strongly associated with the application of a standard/heavy mesh, and it was thus significantly more frequent in patients subjected to Trabucco’s technique. At the one-year follow-up, pain was not related to any patient parameter.

With respect to postoperative hospital stay, a time until discharge longer than 12 h was associated with Trabucco’s or Tisseel fixation techniques, advanced age, presence of comorbidities, cardiovascular and respiratory diseases, ASA score 3, larger hernia size, and antithrombotic therapy (see Table 3). However, all patients that remained in the hospital for more than 24 h underwent Trabucco’s technique (n = 19).

### 3.4. Regression Analysis

A logistic regression analysis was conducted to estimate the impact of surgical treatments on postoperative hematomas, acute pain, or time until discharge, adjusting for potential confounders. Since the intervention was highly related to the type of mesh applied, separate regression analyses were conducted with mesh type as the exposure variable. The results obtained indicated that the odds ratio (OR) for hematoma formation increased as a result of standard/heavy mesh use or Trabucco’s technique, and was also associated with larger hernia size and antiplatelet therapy (see Table 4).

When the postoperative acute pain was set as the outcome, repair with ProGrip and Tisseel glue were both associated with a significantly lower OR compared to the Trabucco’s technique (see Table 5). Similarly, in the analysis conducted with the mesh weight among the covariates, the use of a standard/heavy mesh was found to be an independent risk factor for acute pain. As observed in the bivariate analysis, and regardless of the type of surgery, the greater hernia size had a protective effect from pain, while hematoma increased the pain risk.

Regression analysis for the hospital stay time was conducted excluding interventions, because patients operated on with ProGrip were all discharged within 12 h. The results showed that antithrombotic therapy, older age, and greater hernia size are independent risk factors for overnight stay in hospital (see Table 6).

## 4. Discussion

Ideally, a reliable comparison of different hernioplasty procedures should take into consideration all the patient-specific risk factors which may affect the outcome in the short, medium, and long term. This requirement is even more evident when relatively similar techniques are compared, as in the case of different mesh fixation methods in an open sutureless groin hernia repair. In the present study, postoperative complications and pain were examined for their correlation both with the surgical techniques selected and with several clinical parameters. Most of the latter have been little studied in patients operated on with next-gen sutureless techniques, and knowledge produced so far is still scarce and partly discordant, as in the case of the antithrombotic therapy.

Perioperative administration of antithrombotic drugs implies an increased hazard of intra- and postoperative haemorrhage, especially in the elderly. In low bleeding risk procedures, such as abdominal hernia repair, perioperative withdrawal of antiplatelet agents is generally considered unnecessary. Instead, management of anticoagulants is more challenging because they are mostly indicated for patients with atrial fibrillation, venous thromboembolism, or prosthetic heart valves. Based on a prior assessment of the risk–benefit ratio, physicians may bridge vitamin K antagonists and non-vitamin K oral anticoagulants with short-acting blood thinners or choose not to discontinue direct oral anticoagulants in minor surgeries.

The risk of complications after hernia repair in relation to antithrombotic therapy has been investigated in a few studies, which were mainly focused on interventions entailing extensive dissection, i.e., pre-peritoneal (TEP) or trans-peritoneal (TAPP) laparoscopy. Although the treatment schedule differed in the type of drugs administered and suspension time between studies, most of them reported no differences with respect to wound infection, seroma, hematoma, and/or hernia recurrence between control and treated patients [29,30,31,32,33,34,35]. However, large-case surveys documented a higher frequency of postoperative bleeding and perioperative complications in patients taking antithrombotic therapy [36,37,38]. A retrospective work compared 4870 patients on or off antithrombotic therapy with similar backgrounds, selected through propensity score matching. The results obtained showed a significantly higher rate of postherniorrhaphy haemorrhage in patients receiving antithrombotic medications compared to the control group [37]. Similarly, a multivariate analysis including more than 82,000 patients from the Herniamed Hernia Registry reported a fourfold risk of postoperative secondary bleeding in those who had undergone endoscopic surgery while receiving antithrombotic drugs. In this work, additional factors affecting postoperative bleeding were identified, including coagulopathy, open surgery, older age, higher ASA score, recurrence, male gender, and a large hernia defect [38]. Another retrospective study examined a series of parameters potentially influencing the perioperative outcome in patients undergoing inguinal hernia repair filed in the Herniamed Registry. The overall complication rate displayed a direct correlation with recurrent hernias, bilateral repair, older age, ASA score 3/4, femoral herniation, diabetes, chronic obstructive pulmonary disease (COPD), smoking, antithrombotic drugs and corticosteroids, as well as an inverse correlation with laparoscopic techniques, small hernia defects, female gender, normal body weight, and medial hernia [36].

In agreement with these findings, we reported that hematomas were significantly associated with older age, ASA score 3, male gender, hernia size 3, antiplatelet/anticoagulant therapy, and presence of comorbidities—namely cardiovascular, metabolic, and autoimmune diseases—but also with the use of standard/heavy mesh. However, in our patients, respiratory diseases were not related to postoperative complications, both as a whole and considering only the subgroup of patients with COPD.

Of note, the few cases of infection were all found after surgical interventions using Trabucco’s technique. In the multivariate analysis, beside standard/heavy mesh or Trabucco’s repair, antiplatelet therapy and large hernia size were additional independent risk factors for the onset of hematomas. Differently, the time until discharge was not significantly impacted by the mesh fixation methods, while antithrombotic therapy, older age, and large hernia size increased the OR for admission to inpatient care.

Apart from major complications and recurrence, chronic postoperative inguinal pain (CPIP) is the worst consequence of hernia surgery, impacting the daily life of 0.5% to 6.0% of patients [39]. Persistence of pain has a multifactorial etiology linked to the pain source, surgical procedure type, state of health, and sensitivity of the patient. Moreover, pain may arise from neuropathic, non-neuropathic, visceral, or somatic causes that are often difficult to distinguish from each other and sometimes overlap [40]. By reviewing information available in the literature, Bjurstrom et al. built a comprehensive list of pre-, peri- and postoperative risk factors for chronic postherniorrhaphy inguinal pain which included, among others, heavyweight mesh, postoperative high early pain intensity, and complications [41]. Later on, two large-case studies examined the onset of CPIP in male patients who had undergone primary unilateral inguinal hernia repair, selected from the Herniamed Registry, and both studies concluded that smaller inguinal hernia is another independent risk factor for the development of CPIP [42,43].

In our study, all three techniques considered had a good upshot in terms of pain duration, since in the 30-day follow-up the total percentage of patients with pain was below 2%. In the multivariate analysis, Trabucco’s technique or standard/heavy mesh, small hernia size, and occurrence of hematomas increased the OR of postoperative acute pain, and these findings reflect the risk factors described by previous works for chronic pain. Conversely, the small subgroup of patients reporting mild pain after 1 year (0.9%) did not differ significantly from the others with respect to clinical features, mesh weight, or surgical intervention type. However, this result may be due to the low number of cases available for this outcome (lack of statistical power).

Based on our results, the effect of the hernia size on postoperative acute pain is dual. On the one hand, a larger hernia is associated with a greater incidence of hematomas, which, in turn, increase the OR of pain; on the other hand, a small hernia represents an independent risk factor for pain. With regard to the type of surgical intervention, all repairs employing a standard/heavy mesh were performed using the Trabucco’s technique, which also implies the use of a polypropylene plug. Hence, it can be presumed that the rigidity of these structures is responsible for most cases, albeit infrequent, of postsurgery acute pain and hematoma observed in patients. It is worth noting that the Trabucco’s procedure appears to influence the risk of postoperative acute pain both directly and indirectly by enhancing the risk of hematomas.

To our knowledge, this is the first attempt to compare the performances of Trabucco’s technique, self-gripping mesh, and mesh fixation with glue on patients subjected to open inguinal hernioplasty.

Evidently, our work does not escape the limitations inherent to retrospective multicentre studies, such as the inequality of numbers of patients and surgical interventions performed with the different techniques between surgical units which represents a potential source of bias. In addition, the postoperative complication rate for inguinal hernias is generally low (8–10%) [44] and, therefore, a much greater number of cases would be needed to achieve a high statistical power. Another weak point is the assessment of recurrences with a short-term follow-up, due to the fact that after the first year many patients did not show up for the scheduled checks. Nonetheless, this study presents a fairly substantial number of cases and the results obtained for hernia size and antithrombotic therapy are comparable to those reported by larger-scale surveys, thus supporting the reliability of our analysis.

Overall, Trabucco’s technique displayed a higher incidence of hematomas and postoperative acute pain, as well as a longer hospitalization time compared to ProGrip and the use of Tisseel glue. Moreover, from the analysis of clinical parameters, a non-optimal management of the antithrombotic therapy emerged, which led to an increased OR of postoperative bleeding.

Even if more in-depth and extensive research is needed, we believe that these observations can represent a useful contribution to guide surgeons in choosing surgical and therapeutic strategies tailored to the specific patient conditions. In particular, the opportunity to opt for an alternative sutureless technique to the Trabucco’s method should be considered for patients presenting a large hernia and undergoing antithrombotic therapy.

## Figures and Tables

**Table 1:** Patients’ data summary.

Sex	962 (93.0%) males
	72 (7.0%) females
Age range (years)	18–92
Mean ± SD	60.4 ± 13.6
Hernia location	544 (52.6%) L
	312 (30.2%) M
	157 (15.2%) LM
	20 (2.0%) n.d.
Hernia size	104 (10.1%) (≤1 finger)
	469 (45.4%) (1–2 fingers)
	327 (31.6%) (≥3 fingers)
	134 (13.0%) n.d.
Comorbidities	351 (33.9%) cardiovascular diseases
	77 (7.4%) respiratory diseases
	69 (6.7%) metabolic diseases
	22 (2.1%) autoimmune diseases
	19 (1.8%) infections
N° comorbidities	349 (33.8%) (n = 1)
	89 (8.6%) (n = 2)
	7 (0.7%) (n = 3)
Antithrombotic therapy	872 (84.3%) none
	121 (11.7%) antiplatelet agents
	41 (4.0%) anticoagulants
ASA score	880 (85.1%) (1–2)
	81 (7.8%) (3)
	73 (6.9%) n.d.
Intervention	537 (51.9%) Trabucco
	312 (30.2%) ProGrip
	185 (17.9%) Tisseel fixation
Mesh weight	497 (48.1%) Light
	537 (51.9%) Standard/Heavy
Time until discharge	883 (85.3%) (≤12 h)
	151 (14.6%) (>12 h)

L, lateral/indirect; M, medial/direct; LM, latero-medial; n.d., not determined.

**Table 2:** Bivariate analysis for postoperative complications.

	Hematoma	*p*-Value	Scrotal Edema	*p*-Value	Seroma	*p*-Value	Infection	*p*-Value
**Age (yrs) median (IQR)**		**<0.001**		0.865		0.565		0.674
No	61.0 (19.0)		60.2 (18.0)		61.0 (19.0)		61.0 (19)	
Yes	70.0 (15.0)		62.2 (19.0)		58.0 (17.0)		68.0 (21.5)	
**Gender**		**0.007**		0.303		0.55		0.45
F	0 (0.0%)		1 (1.4%)		1 (1.4%)		0 (0.0%)	
M	67 (7.0%)		4 (0.4%)		10 (1.0%)		11 (1.1%)	
**Hernia location**		0.264		0.451		0.086		0.541
L	37 (6.8%)		2 (0.4%)		10 (1.8%)		5 (0.9%)	
M	14 (8.9%)		3 (1.0%)		1 (0.6%)		3 (1.0%)	
LM	15 (4.8%)		0 (0.0%)		1 (0.6%)		3 (1.9%)	
**Hernia size**		**<0.001**		0.424		0.085		0.494
1	1 (1.0%) _a_		1 (1.0%)		2 (1.9%)		1 (1.0%)	
2	23 (4.9%) _a_		3 (0.6%)		2 (0.4%)		2 (0.4%)	
3	35 (10.7%) _b_		1 (0.3%)		6 (1.8%)		3 (0.9%)	
**CVD**		**<0.001**		0.45		0.758		0.11
No	30 (4.4%)		4 (0.6%)		8 (1.2%)		10 (1.5%)	
Yes	37 (10.5%)		1 (0.3%)		3 (0.9%)		1 (0.3%)	
**Respiratory diseases**		0.333		0.679		0.195		0.575
No	60 (6.3%)		5 (0.5%)		9 (0.9%)		10 (1.0%)	
Yes	7 (9.1%)		0 (0.0%)		2 (2.6%)		1 (1.3%)	
**Metabolic diseases**		**0.01**		0.292		0.534		0.466
No	57 (5.9%)		4 (0.4%)		10 (1.0%)		11 (1.1%)	
Yes	10 (14.5%)		1 (1.4%)		1 (1.4%)		0 (0.0%)	
**Autoimmune diseases**		**0.049**		0.898		0.788		0.788
No	63 (6.2%)		5 (0.5%)		11 (1.1%)		11 (1.1%)	
Yes	4 (18.2%)		0 (0.0%)		0 (0.0%)		0 (0.0%)	
**Infectious diseases**		0.629		0.911		0.815		0.815
No	67 (6.6%)		5 (0.5%)		11 (1.1%)		11 (1.1%)	
Yes	0 (0.0%)		0 (0.0%)		0 (0.0%)		0 (0.0%)	
**Comorbidities**		**<0.001**		1		1		0.359
0	23 (3.9%) _a_		3 (0.5%)		6 (1.0%)		9 (1.5%)	
1	30 (8.6%) _b_		2 (0.6%)		4 (1.1%)		2 (0.6%)	
2	12 (13.5%) _b_		0 (0.0%)		1 (1.1%)		0 (0.0%)	
3	2 (28.6%) _b_		0 (0.0%)		0 (0.0%)		0 (0.0%)	
**ASA score**		**<0.001**		0.643		0.622		0.622
1–2	48 (5.5%)		5 (0.6%)		10 (1.1%)		10 (1.1%)	
3	14 (17.3%)		0 (0.0%)		1 (1.2%)		1 (1.2%)	
**Therapy**		**<0.001**		0.574		0.055		1
None	37 (4.2%) _a_		4 (0.5%)		7 (0.8%) _a_		10 (1.1%)	
Antiplatelets	24 (19.8%) _b_		1 (0.8%)		4 (3.3%) _b_		1 (0.8%)	
Anticoagulants	6 (14.6%) _b_		0 (0.0%)		0 (0.0%) _a,b_		0 (0.0%)	
**Intervention**		0.066		0.12		0.126		**0.004**
ProGrip	14 (4.5%)		0 (0.0%)		4 (1.3%)		0 (0.0%) _a_	
Trabucco	44 (8.2%)		5 (0.9%)		3 (0.6%)		11 (2.0%) _b_	
Tisseel	9 (4.9%)		0 (0.0%)		4 (2.2%)		0 (0.0%) _a,b_	
**Mesh**		**0.02**		**0.037**		0.131		**0.001**
Light	23 (4.6%)		0 (0.0%)		8 (1.6%)		0 (0.0%)	
Standard/Heavy	44 (8.2%)		5 (0.9%)		3 (0.6%)		11 (2.0%)	

Number and percentage of events are shown for each category, together with probabilities. IQR, interquartile range; CVD, cardiovascular diseases. Categories that differ significantly are marked with different subscripts (a or b). *p*-values under the 0.05 threshold are highlighted in bold.

**Table 3:** Bivariate analysis for postoperative acute and chronic pain, and time until discharge.

	Acute Pain	*p*-Value	Chronic Pain	*p*-Value	Time until Discharge	*p*-Value
					>12 h	
**Age: median (IQR)**		0.669		0.831		**<0.001**
No	61 (21.0)		57.5 (24.7)		59.4 (18.0)	
Yes	61 (19.0)		61 (15.7)		64.5 (19.5)	
**Gender**		0.631		0.545		0.863
F	14 (19.4%)		0 (0.0%)		11 (15.3%)	
M	167 (17.4%)		9 (1.0%)		140 (14.6%)	
**Hernia location**		0.441		0.193		0.456
L	106 (19.5%)		5 (1.0%)		89 (16.4%)	
M	48 (15.4%)		1 (0.3%)		43 (13.8%)	
LM	27 (17.2%)		3 (1.9%)		19 (12.1%)	
**Hernia size**		**<0.001**		1		**0.009**
1	32 (30.8%) _a_		1 (1.2%)		10 (9.6%) _a,b_	
2	98 (20.9%) _a_		5 (1.2%)		60 (12.8%) _b_	
3	45 (13.8%) _b_		3 (1.0%)		64 (19.6%) _a_	
**Cardiovascular diseases**		0.3		0.644		**0.033**
No	126 (18.4%)		6 (0.9%)		88 (12.9%)	
Yes	55 (15.7%)		3 (0.9%)		63 (17.9%)	
**Respiratory diseases**		0.534		0.146		**0.017**
No	170 (17.8%)		7 (0.8%)		132 (13.8%)	
Yes	11 (14.3%)		2 (2.7%)		19 (24.7%)	
**Metabolic diseases**		0.412		0.545		0.292
No	172 (17.8%)		9 (1.0%)		138 (14.3%)	
Yes	9 (13.0%)		0 (0.0%)		13 (18.8%)	
**Autoimmune diseases**		0.783		0.818		0.549
No	178 (17.6%)		9 (1.0%)		147 (14.5%)	
Yes	3 (13.6%)		0 (0.0%)		4 (18.2%)	
**Infectious diseases**		0.759		0.15		0.179
No	177(17.4%)		8 (0.9%)		146 (14.4%)	
Yes	4 (21.1%)		1 (5.9%)		5 (26.3%)	
**Comorbidities**		0.343		0.452		**<0.001**
0	111 (18.8%)		4 (0.7%)		66 (11.3%) _a_	
1	59 (16.9%)		5 (1.6%)		63 (18.1%) _b_	
2	11 (12.4%)		0 (0.0%)		18 (20.2%) _a,b_	
3	0 (0.0%)		0 (0.0%)		4 (57.1%) _b_	
**ASA score**		0.172		0.495		**0.037**
1–2	133 (15.1%)		8 (1.0%)		132 (15.0%)	
3	16 (19.8%)		0 (0.0%)		19 (23.5%)	
**Therapy**		0.904		0.122		**<0.001**
None	155 (17.8%)		6 (0.7%)		97 (11.1%) _a_	
Antiplatelets	20 (16.5%)		3 (2.8%)		31 (25.6%) _b_	
Anticoagulants	6 (14.6%)		0 (0.0%)		23 (56.1%) _c_	
**Intervention**		**<0.001**		0.162		**<0.001**
ProGrip	31 (9.9%) _a_		4 (1.4%)		0 (0.0%) _a_	
Tisseel	10 (5.4%) _a_		3 (1.7%)		64 (34.6%) _b_	
Trabucco	140 (26.1%) _b_		2 (0.4%)		87 (16.2%) _c_	
**Mesh**		**<0.001**		0.105		0.135
Light	41 (8.2%)		7 (1.5%)		64 (12.9%)	
Standard/Heavy	140 (26.1%)		2 (0.4%)		87 (16.2%)	
**Hematoma**		**<0.001**		0.076		0.473
No	148 (15.3%)		7 (0.8%)		139 (14.4%)	
Yes	33 (49.3%)		2 (4.0%)		12 (17.9%)	
**Scrotal edema**		**<0.001**				0.453
No	176 (17.1%)		-		151 (14.7%)	
Yes	5 (100%)				0 (0.0%)	
**Seroma**		0.109		0.073		0.668
No	177 (17.3%)		8 (0.8%)		149 (14.6%)	
Yes	4 (36.4%)		1 (12.5%)		2 (18.2%)	
**Infection**		0.7		0.901		0.506
No	180 (17.6%)		9 (1.0%)		150 (14.7%)	
Yes	1 (9.1%)		1 (0.0%)		1 (9.1%)	

Number and percentage of events are shown for each category, together with probabilities. IQR, interquartile range. Categories that differ significantly are marked with different subscripts (a, b, or c). *p*-values under the 0.05 threshold are highlighted in bold.

**Table 4:** Multivariate analysis for hematomas.

	Logistic Regression Including Interventions		Logistic Regression Including Mesh Weight	
	Hematomas		Hematomas	
	Odds Ratio (95% CI)	*p*-Value	Odds Ratio (95% CI)	*p*-Value
**Intervention**				
Trabucco	1			
ProGrip	0.56 (0.27–1.14)	0.109	-	-
Tisseel	0.37 (0.16–0.84)	**0.018**		
**Mesh**				
Light	-	-	1	
Standard/Heavy			2.14 (1.17–3.92)	**0.014**
**Age**	1.02 (0.99–1.05)	0.221	1.02 (0.99–1.05)	0.252
**Cardiovascular diseases**				
No	1		1	
Yes	1.28 (0.65–2.52)	0.474	1.30 (0.66–2.55)	0.451
**Autoimmune diseases**				
No	1			
Yes	0.83 (0.10–6.99)	0.866	-	-
**Hernia size**				
1	1		1	
2	5.17 (0.67–39.72)	0.115	4.97 (0.65–38.17)	0.123
3	9.92 (1.30–75.85)	**0.027**	9.51 (1.25–72.55)	**0.03**
**Therapy**				
None	1		1	
Antiplatelets	4.95 (2.45–10.00)	**<0.001**	4.69 (2.35-9.33)	**<0.001**
Anticoagulants	1.72 (0.50–5.92)	0.39	1.63 (0.48-5.56)	0.436
**ASA score**				
1–2	1		1	
3	0.81 (0.37–2.17)	0.807	0.95 (0.39–2.27)	0.902

CI, confidence interval. *p*-values under the 0.05 threshold are highlighted in bold.

**Table 5:** Multivariate analysis for postoperative acute pain.

	Logistic Regression Including Interventions		Logistic Regression Including Mesh Weight	
	Postoperative Acute Pain		Postoperative Acute Pain	
	Odds Ratio (95% CI)	*p*-Value	Odds Ratio (95% CI)	*p*-Value
**Intervention**				
Trabucco	1			
ProGrip	0.27 (0.18–0.42)	**<0.001**	-	-
Tisseel	0.13 (0.07–0.27)	**<0.001**		
**Mesh**				
Light	-	-	1	
Standard/Heavy			4.56 (3.07–6.76)	**<0.001**
**Hernia size**				
1	1		1	
2	0.63 (0.38–1.03)	0.066	0.60 (0.36–0.98)	**0.043**
3	0.31 (0.17–0.55)	**<0.001**	0.29 (0.16–0.53)	**<0.001**
**Hematoma**				
No	1		1	
Yes	7.55 (4.12–13.86)	**<0.001**	7.47 (4.08–13.61)	**<0.001**

CI, confidence interval. *p*-values under the 0.05 threshold are highlighted in bold.

**Table 6:** Multivariate analysis for the time to discharge.

	Time until Discharge > 12 h	
	Odds Ratio (95% CI)	*p*-Value
**Mesh**		
Light	1	
Standard/Heavy	1.47 (0.98–2.22)	0.065
**Age**	1.02 (1.00–1.04)	**0.014**
**Hernia size**		
1	0.48 (0.22–1.01)	0.053
2	0.64 (0.43–0.97)	**0.035**
3	1	
**Cardiovascular diseases**		
No	1	
Yes	0.63 (0.40–1.00)	0.053
**Respiratory diseases**		
No	1	
Yes	1.49 (0.79–2.82)	0.221
**Therapy**		
None	1	
Antiplatelets	2.38 (1.38–4.10)	**0.002**
Anticoagulants	8.53 (3.77–19.27)	**<0.001**
**ASA score**		
1–2	1	
3	0.48 (0.22–1.05)	0.065

CI, confidence interval. *p*-values under the 0.05 threshold are highlighted in bold.

## Data Availability

The data presented in this study are available on request from the corresponding author.
